# Cigarette Prices and Smoking Among Youth in 16 African Countries: Evidence From the Global Youth Tobacco Survey

**DOI:** 10.1093/ntr/ntac017

**Published:** 2022-01-17

**Authors:** Samantha Filby, Corne van Walbeek

**Affiliations:** Research Unit on the Economics of Excisable Products (REEP), School of Economics, University of Cape Town, Cape Town, South Africa; Research Unit on the Economics of Excisable Products (REEP), School of Economics, University of Cape Town, Cape Town, South Africa

## Abstract

**Introduction:**

African countries have among the lowest excise taxes in the world. This paper provides new evidence on the association between cigarette prices and youth smoking in 16 African countries.

**Aims and Methods:**

We use Global Youth Tobacco Survey (GYTS) cross-country data from approximately 67 500 participants. The relationship between prices and youth smoking in Africa is estimated using probit models for smoking participation and generalized linear models for conditional cigarette demand. Each model is estimated using local-brand and foreign-brand cigarette prices.

**Results:**

Higher prices are associated with lower demand across African countries, for both smoking prevalence and the intensity of cigarette consumption by smokers. The estimated price elasticity of participation is −0.70 [95% CI: −1.28 to −0.12] for local-brand cigarettes and −0.71 [95% CI: −0.98 to −0.44] for foreign-brand cigarettes. The price elasticity of conditional cigarette demand is −0.44 [95% CI: −0.76 to −0.12] for local brands and −0.75 [95% CI: −0.96 to −0.53] for foreign brands. The total price elasticity of demand for youth in our sample is −1.14 for local brands and −1.46 for foreign brands.

**Conclusions:**

Higher cigarette prices significantly decrease the likelihood of smoking and decrease the intensity of cigarette consumption among African youths. Increases in the excise tax that increase the retail price of cigarettes will play an important role in reducing youth tobacco use on the continent. Governments are encouraged to increase excise taxes in order to improve public health.

**Implications:**

Evidence on the association between cigarette prices and youth smoking in African countries is limited. The Global Youth Tobacco Survey (GYTS) was first introduced in 1999. In 2012, the Centers for Disease Control and Prevention revised the GYTS questionnaire, which removed some questions and introduced new questions into the survey. To the best of our knowledge, there are no published estimates of the relationship between cigarette prices and demand that have used this more recent individual-level GYTS data for African countries. In conducting this analysis, we add to the limited literature on the association between cigarette prices and youth smoking in Africa.

## Introduction

Primarily because of predicted rapid population growth, the number of smokers in Africa is projected to increase to such an extent that scholars have flagged the continent as the future epicenter of the tobacco epidemic.^1^ Because most tobacco users start smoking during their adolescent years,^2^ tobacco-control strategies targeting youth will be essential to prevent a future tobacco epidemic in the region.

A voluminous literature shows that tobacco taxation is an effective means to reduce smoking, especially among the youth.^2,3^ Global-level studies have further shown that youth in low- and middle-income countries (LMICs) are more responsive to cigarette price changes than youth in high-income countries.^4,5^ The policy implication for African countries is that excise taxes should be increased to reduce youth smoking to prevent the onset of the epidemic. Yet, excise tax rates in African countries are among the lowest in the world.^6^

While a growing body of evidence from Africa shows that the demand for cigarettes is price inelastic among adults,^7–16^ only a handful of studies examine the association between cigarette prices and youth cigarette smoking.^17–19^ In addition, the existing evidence is largely focused on the relationship between price, prevalence, and smoking onset, while the impact of tobacco prices on youth smoking intensity remains largely unexplored.

In this study, we use individual-level data on smoking behavior, environments, and attitudes from the Global Youth Tobacco Survey (GYTS) to provide new evidence on the association between cigarette prices, smoking participation, and conditional cigarette demand (ie, intensity) among youth living in selected African countries.

To the best of our knowledge, there are no published estimates of the relationship between cigarette prices and demand that have taken advantage of the more recent individual-level African GYTS data. In conducting this analysis, we add to the very limited literature on the association between cigarette prices and youth smoking in Africa.

## Methods

### Data

The GYTS is a nationally representative, school-based survey that employs a standardized methodology to track tobacco use among young people across countries.^20^ As well as collecting information on cigarette prevalence and consumption, the survey also captures respondents’ basic demographic information and asks about their exposure to confounding factors, such as cigarette advertising, antismoking messaging, and the ease of purchasing cigarettes. The GYTS has been conducted in 45 of the 47 WHO-AFRO countries. Twenty-two of these countries have implemented more than one nationally representative wave of the GYTS.

The GYTS was first introduced in 1999. In 2012, the Centers for Disease Control and Prevention (CDC) revised its GYTS questionnaire protocol, removing some questions and introducing new ones into the survey.^20^ The revision also ensured national representativity of the GYTS surveys as the standard. To avoid complexities surrounding differences in the survey questionnaires administered before and after the revised GYTS protocol, and to make use of the more recent and underutilized GYTS data, we focus only on those African countries that conducted a GYTS after 2012. These countries are listed in Table 1. We further exclude the Comoros from our analysis as no price data are available for this country. Countries marked with an asterisk have more than one nationally representative round of GYTS data available, but the earlier survey was conducted before 2012 and is thus not analyzed further.

**Table 1.** Datasets Available for Countries Implementing the GYTS After the Revised Protocol 2012

Country	Survey year	GYTS sample (*n*)	Population aged 10–19 in millions (*N*)
Algeria	2013	6228	6.10
Cameroon	2014	2922	5.21
The Comoros†	2015		
Gabon	2014	1781	0.37
The Gambia	2017	12 585	0.51
Ghana*	2017	5664	6.29
Kenya*	2013	1895	10.70
Madagascar*	2018	2920	6.11
Mauritania*	2018	3740	0.96
Mauritius*	2016	4141	0.19
Mozambique	2013	5599	6.08
Senegal*	2013	1728	3.16
The Seychelles*	2015	2485	0.01
Sierra Leone	2017	6680	1.74
Tanzania	2016	3840	12.25
Uganda*	2018	3458	10.68
Zimbabwe	2014	1780	3.13

^†^The Comoros is excluded from our analysis because of the unavailability of price data.

While the 16 countries included in our analysis provide the most recent (and thus policy-relevant) data from the GYTS, a drawback of using these data is that many of the countries implementing a GYTS after 2012 only have data for 1 year, rather than multiple cross-sections of data over time (Table 1). This limits our ability to control for country-fixed effects that could influence the relationship between prices and smoking. We therefore cannot use these data to establish causality between price and smoking behavior for our sample of countries.

In an analysis of cigarette prices and smoking among adults in 13 non-African LMICs, Kostova et al. demonstrate that in a pooled country cross-sectional framework, one can use between-country price variation to estimate the direction of the relationship between prices and smoking outcomes.^21^ In their study, unobserved country differences are proxied by GDP per capita, average local rates of exposure to cigarette advertising, and average local rates of exposure to antismoking messaging.

In the current paper, where each country is also represented by a single year of data, we use the difference in cigarette prices across countries to study the association between cigarette prices, smoking participation, and smoking intensity using Cragg’s two-part model of cigarette demand.^22^ To account for the fact that the GYTS sample size “oversamples” some countries relative to the actual size of their young populations, and “under-samples” others, our empirical specification, elaborated on in the section “Empirical Specification”, uses weighted data, where we calculate a weight for each country, as:


$$\begin{array}{l} Weight_{i}\;=\;\;\;\;\frac{\overset{i=16}{\underset{i=1}{\sum }}n_{i}}{n_{i}}\;\times \;\frac{N_{i}}{\overset{i=16}{\underset{i=1}{\sum }}N_{i}} \end{array}$$ [1]

where *n*_*i*_ is the sample size of country *i*, and *N*_*i*_ is the size of the youth population in country *i*. Ideally, the youth population age group should correspond to the age group of the sample (ie, 13–17). However, these data were not available in the World Bank Development Indicators data, and thus we approximated the population with all youth aged 10–19.

### Dependent Variables

The first regression in the two-part model estimates an individual’s decision to smoke.^23^ The dependent variable, which is taken from the GYTS, takes a value of 1 if individuals indicated that they smoked cigarettes on 1 or more days in the past month, and 0 if they did not smoke in the past month. Respondents not reporting their smoking status (*n* = 5282, 7.3% of observations) are not included in the model. The highest smoking prevalence is observed in Zimbabwe (17.81%) and the lowest smoking prevalence in Tanzania (1.26%) (Table 2).

**Table 2.** Sample Means

Country	Survey year	% Current smokers	Average number of cigarettes smoked per month per smoker	Age	% Male	% Receiving pocket money	% With at least one parent that smokes	Number of observations	Adult smoking prevalence (%)
Algeria	2013	9.1	118.1	14.8	47.1	78.7	29.8	6049	14.0
Cameroon	2014	6.6	53.4	14.2	53.1	78.0	15.9	2768	5.0
Gabon	2014	9.1	37.1	15.0	49.0	78.9	15.5	1708	–
The Gambia	2017	8.5	19.4	14.8	47.3	88.2	24.5	11 589	9.0
Ghana	2017	3.0	60.1	14.2	50.4	89.6	10.0	5213	2.0
Kenya	2013	5.0	24.4	14.4	50.8	51.7	13.2	1818	7.0
Madagascar	2018	11.2	33.0	14.4	48.0	78.6	18.9	2580	13.0
Mauritania	2018	13.2	92.6	14.0	51.7	77.4	25.1	3252	7.0
Mauritius	2016	15.2	49.2	13.9	48.4	84.5	31.9	3970	15.0
Mozambique	2013	2.3	27.8	15.1	52.8	72.1	7.4	5086	11.0
Senegal	2013	5.6	45.1	14.7	53.0	56.8	12.7	1619	5.0
The Seychelles	2015	15.6	42.7	13.9	49.2	93.6	31.0	2312	15.0
Sierra Leone	2017	4.0	25.7	15.1	50.0	60.9	23.3	6253	12.0
Tanzania	2016	1.3	68.9	14.0	48.4	58.7	9.1	3627	6.0
Uganda	2018	4.1	13.5	14.9	50.6	68.3	8.2	3329	5.0
Zimbabwe	2014	17.8	155.5	14.0	48.6	69.5	25.0	5553	8.0

Means derived using weights for complex survey design.

The second part of the two-part model examines the number of cigarettes consumed by smokers (conditional cigarette demand).^23^ We measure the dependent variable—smoking intensity (consumption)—based on the average number of days that smoking occurred in the past month multiplied by the average number of cigarettes smoked on a smoking day, as reported by each respondent in the GYTS for each country. Weighted average cigarette consumption among smokers in our sample is 59.6 cigarettes per month, ie, approximately two cigarettes per day. Average cigarette consumption is highest in Zimbabwe (155.5 cigarettes per month), and lowest in Uganda (13.5 cigarettes per month) (Table 2). In the following subsection, we outline and discuss each of the control variables included in the two regressions.

### Independent Variables

We employ three types of independent variables in our regressions analysis: individual-level variables, primary sampling unit (PSU)-level variables, and country-level variables.

#### Individual-Level Variables

Our individual-level variables are taken from the GYTS. We control for age, age squared, school grade, gender, income (“pocket money”), and parental smoking. We present the sample means of these variables in Table 2 and discuss the construction of each of these variables below.

##### Age.

The GYTS asks the age of all individuals who are surveyed. We also include age squared as a control in both regressions. Ages range between 11 and 17. The average respondent in our sample is 14.4 years old.

##### Grade.

We add grade dummies in addition to age to account for the influence of peer groups on smoking outcomes. The number of grades sampled varies by country. The minimum number of grades sampled is 3, the maximum number of grades sampled is 5. We create a categorical variable equal to 1 for the lowest grade (which we call grade 7) and equal to 5 for the highest grade (which we call grade 11).

##### Gender.

We create a dichotomous indicator equal to 1 for males and 0 for females. Our sample is divided equally between males (50.0%) and females (50.0%).

##### Availability of Pocket Money.

Availability of pocket money is a binary indicator equal to 1 if the individual receives pocket money, and 0 otherwise. This serves as a proxy for access to money as GYTS does not ask any questions about the income of respondents. About 69% of respondents in our sample receive pocket money. The Gambia has the highest proportion of students receiving pocket money (88.2%), while Kenya has the lowest (51.7%) (Table 2).

##### Parental Smoking.

Parental smoking is defined by a binary indicator equal to 1 if the individual has a parent who smokes. Cameroon, Gabon, and Senegal did not ask students about parental smoking directly but provided suitable proxies. In these three countries, students were asked: “During the past 7 days, on how many days has anyone smoked inside your home, in your presence?” and given options of 0 days, 1–2 days, 3–4 days, 5–6 days, or 7 days. We coded parental smoking equal to 1 if the student had someone who smoked in their presence in their home for 3–4 days or more. Approximately 15% of respondents in the sample have at least one parent who smokes. Mauritius has the highest proportion of students with at least one parent who smokes (31.9%), followed by the Seychelles (31.0%). The country with the lowest proportion of students with at least one parent who smokes is Mozambique (7.4%) (Table 2). Using adult daily smoking prevalence data from the WHO Report on the Global Tobacco Epidemic, shown in Table 2, we find a positive correlation coefficient of 0.71 between adult smoking prevalence and the percentage of parents who smoke, for our sample of countries.

#### PSU-Level Variables

We include four variables that control for the local tobacco-related environment: (1) a proxy for local antismoking sentiment, (2) a measure of local rates of exposure to cigarette advertising, (3) a measure of local rates of exposure to antismoking messaging, and (4) a measure of the ease of purchasing cigarettes. All variables are constructed by aggregating and/or averaging student responses at the PSU level to reduce potential endogeneity of individual responses.

Although we intend these PSU-level controls to capture local-level differences, they do not necessarily proxy for the underlying tobacco-control policies (eg, the fact that a larger proportion of students who are denied buying cigarettes because of their age does not necessarily imply better enforcement of age restriction laws). This is because most countries in our sample had not implemented comprehensive tobacco-control programs by the time the GYTS surveys were conducted. Table 3 presents the summary statistics of these PSU-level variables.

**Table 3.** Means of Country-Level and PSU-Level Variables

Country-level					PSU-level				
Country	% Living below the poverty line	% Living in urban areas	Local-brandPPP adjusted constant 2017 dollars	Foreign-brandPPP adjusted constant 2017 dollars	Average PSU-level advertising exposure rate	Average PSU-level exposure rate to antitobacco messaging	Average PSU-level antismoking sentiment	Average PSU-level ease of access to cigarettes	Number of PSUs
Algeria	0.4	70	2.7	4.6	0.66	0.73	0.92	0.22	43
Cameroon	26.0	54	1.5	5.4	0.70	0.70	0.77	0.32	25
Gabon	3.4	88	2.1	3.7	0.65	0.75	0.83	0.23	24
Gambia	10.3	61	2.6	4.7	0.50	0.52	0.81	0.45	57
Ghana	12.7	55.4	1.4	5.6	0.52	0.59	0.67	0.41	26
Kenya	37.1	25	1.7	5.6	0.65	0.71	0.46	0.22	25
Madagascar	78.8	37.2	1.6	5.6	0.72	0.72	0.94	0.30	26
Mauritania	6.0	53.7	1.2	7.9	0.54	0.67	0.66	0.45	25
Mauritius	0.2	41	6.6	6.9	0.58	0.71	0.67	0.42	75
Mozambique	63.7	33	1.6	5.1	0.63	0.65	0.81	0.41	19
Senegal	38.5	45	2.5	4.3	0.49	0.81	0.76	0.31	35
The Seychelles	1.2	55	13.0	16.1	0.64	0.64	0.72	0.39	118
Sierra Leone	54.7	42	1.8	2.2	0.63	0.64	0.82	0.34	25
Tanzania	49.4	32	3.6	–	0.52	0.65	0.76	0.48	26
Uganda	41.3	23.8	2.0	6.7	0.58	0.63	0.77	0.48	26
Zimbabwe	33.9	23	2.3	3.8	0.65	0.59	0.49	0.40	26

PPP, purchasing power parity; PSU, primary sampling unit.

Local prevalence of cigarette advertising exposure is calculated as the proportion of respondents who have recently (in the last 30 days) been exposed to people using tobacco on television, in videos, or in movies, or to advertisements or promotions for tobacco products at points of sale (eg, at shops, kiosks, etc.). Local prevalence of antitobacco media campaigns is the proportion of respondents who have recently (in the last 30 days) been exposed to antismoking messages in broadcast and print media or at gatherings such as sports events or fairs. Ease of access to cigarettes is calculated as the proportion of survey participants who have recently tried to purchase cigarettes but were denied the purchase by vendors because of their age. Antismoking sentiment is defined as the percentage of non-smokers who favor bans on smoking in public places. As is standard practice, smokers are excluded from the construction of this variable because smokers are disproportionately more likely than non-smokers to disapprove of smoking bans.^4,5^

#### Country-Level Variables

Our country-level variables are cigarette prices, the proportion of people living below the poverty line, and the proportion of people living in urban areas. Means of the country-level variables are presented in Table 3. We discuss the construction of each of the country-level variables included in our analysis below.

##### Price.

We run our regressions for two different prices: a cheap price (proxied by a local-brand price) and an expensive price (proxied by a foreign-brand price). Price data are obtained from countries’ implementation reports to the WHO FCTC Convention Secretariat,^24^ and various years of the WHO’s Report on the Global Tobacco Epidemic.^6^

Every odd-numbered year (eg, 2017), the WHO’s Report on the Global Tobacco Epidemic releases retail price data for a pack of 20 cigarettes of a premium brand (Marlboro, or the nearest international equivalent) and the cheapest brand in a country in the previous even-numbered year. Price data are collected in the capital city of each country, in local currency.^25^ These prices are collected at two different types of outlets: high-volume supermarkets and smaller retail outlets.^25^ Price data for Cameroon, Gabon, Madagascar, Mauritania, Mauritius, Tanzania, Uganda, and Zimbabwe are taken from the biennial WHO report on the Global Tobacco Epidemic. For all these countries, we found data on the cheapest cigarette brand and the most expensive cigarette brand in the GYTS survey year. Tanzania only provided information on the price of the most popular brand. We assume that this price is for a local (cheap) brand.

Price data for Algeria, the Gambia, Ghana, Senegal, the Seychelles, and Sierra Leone are taken from submissions to the WHO FCTC Secretariat on their implementation of the WHO FCTC.^24^ These reports are submitted to the Convention Secretariat every 2 years. Countries report in even-numbered years (eg, 2014) on their implementation progress in the previous, odd-numbered, year. The reporting instrument asks countries to provide the retail prices of the three most widely sold brands of both domestic and imported tobacco products at the most widely used outlet in their capital city. To ensure comparability with the WHO’s cheapest and most expensive brands, we take the lowest price of the local brand and the highest price of the imported brand reported by the country, respectively.

We could not find price data that matched the GYTS survey year for Kenya, Mozambique, and the Comoros. While we were able to find price data from the WHO Report on the Global Tobacco Epidemic for the year after each GYTS survey (2014 prices for Mozambique and Kenya and 2016 prices for the Comoros), we were only able to find appropriate Consumer Price Index data for Mozambique and Kenya with which to deflate the 2014 cigarette price to 2013 levels. Because we were unable to do the same for the Comoros, we exclude the Comoros from our analysis.

All prices are adjusted using purchasing power parity (PPP) conversion factors obtained from the World Bank’s World Development Indicators database. Prices enter our models in logarithmic form. Prices expressed in real 2017 PPP dollars are shown in Table 3. The lowest prices for local cigarettes are observed in Mauritania (1.18 2017 PPP dollars); the highest price is observed in the Seychelles (13.00 2017 PPP dollars). For foreign-brand cigarettes, the highest price is observed in the Seychelles (16.10 2017 PPP dollars) and the lowest price is observed in Sierra Leone (2.23 2017 PPP dollars).

##### Poverty Headcount Ratio at PPP$1.90 a Day.

Kostova et al. used per capita GDP as a catchall proxy for unobserved country differences.^21^ This variable gave them a statistically significant coefficient. Provisional specifications with per capita GDP for our sample of countries did not yield a significant coefficient on this variable. Given the high degree of inequality in the countries in our sample, and the fact that per capita GDP is an average measure of well-being, we believe that the percentage of people living below the poverty line in each country is a better measure of standard of living than per capita GDP for the countries in our sample. In order to control for the impact of poverty levels on tobacco use, we include the percentage of the population living on less than $1.90 a day (at 2011 international prices) in our regressions. These data were obtained from the World Bank Development Indicators and serve as a proxy for unobserved country differences. Algeria has the lowest proportion of people living below the poverty line (0.4%), while Madagascar has the highest (78.8%) (Table 3).

##### Proportion of People Living in Urban Areas.

We obtain these data from the World Bank Development Indicators. A number of studies have shown that place of residence (ie, urban or rural) is a significant determinant of both smoking participation and smoking intensity,^26–28^ but the direction of the effect is not clear. Research from Europe^29^ and a group of 13 LMICs^21^ suggests that smoking prevalence is higher in urban areas, while evidence from the United States,^27^ Poland,^28^ and India^26^ shows that rural areas have higher smoking rates than urban areas.

### Empirical Specification

To study the association between cigarette prices, smoking participation, and smoking intensity, we use Cragg’s two-part model of cigarette demand. In part 1, we estimate the models of smoking participation with a probit regression and report on the average marginal effects.^23^ In part 2, based on the most favorable Akaike’s information criterion (AIC) statistic between competing models, we use a generalized linear model with a normal distribution and log link to estimate the covariates of smoking intensity in our sample. The number of cigarettes smoked by smokers enters our model in logarithmic form so that the coefficient on the logarithm of price is interpreted as an intensity (or conditional) elasticity.

For both the smoking participation and intensity models, each specification is estimated using both local-brand and foreign-brand cigarette prices. Models using foreign-brand cigarette prices exclude Tanzania because it did not have data on foreign-brand prices.

Missing data on the independent variables make up less than 1% of observations and so individuals with non-responses on any of the analysis variables were excluded from our analysis. Our final sample consists of 59 447 respondents from 16 countries.

For the regressions, we employ a pooled country cross-sectional framework. For the country-level variables (eg, cigarette prices), each variable has a single value for each country. To account for correlation between smoking outcomes within countries, we cluster standard errors by country and report the price elasticities of smoking participation and conditional cigarette demand at the mean characteristics of the sample.

We calculate the total (or unconditional) price elasticity of demand by adding the price elasticities of demand from the first and second parts of the two-part model (ie, the prevalence price elasticity and the conditional price elasticity of demand).

## Results

### Smoking Participation

Results from the smoking participation models for local-brand prices and foreign-brand prices are presented in the first and second columns of Table 4, respectively. Higher cigarette prices are significantly (at the 5% level for local brands and at the 1% level for international brands) associated with lower smoking prevalence. The estimated price elasticity of participation is −0.70 [95% CI: −1.28 to −0.12] for local-brand cigarettes and −0.71 [95% CI: −0.98 to −0.44] for foreign-brand cigarettes.

**Table 4.** Probit Models of Smoking Participation (Local- and Foreign-Brand Prices)

	Smoking participation	
	Local-brand prices	Foreign-brand prices
	(*N* = 59 447)	(*N* = 55 902)
Log of cigarette price	−0.029** (0.012)	−0.033*** (0.006)
Local rate of exposure to cigarette advertising	0.088*** (0.033)	0.068 (0.192)
Local rate of exposure to antismoking messages	0.006 (0.023)	−0.040*** (0.016)
Antismoking sentiment	−0.020 (0.015)	0.001 (0.013)
Youth access	−0.040*** (0.010)	−0.019* (0.011)
Age	−0.072*** (0.015)	−0.060*** (0.015)
Age2	0.002*** (0.001)	0.002*** (0.001)
Male	0.046*** (0.012)	0.060*** (0.016)
Pocket money	0.023*** (0.004)	0.024*** (0.003)
Parental smoking	0.067*** (0.009)	0.063*** (0.012)
Education (relative to grade 7)		
Grade 8	0.122** (0.053)	0.008* (0.005)
Grade 9	0.016 (0.067)	0.001 (0.011)
Grade 10	0.138 (0.127)	0.004 (0.013)
Grade 11	1.24*** (0.227)	0.093*** (0.019)
% of the population living below the PPP$1.90 poverty line	−0.002 (0.004)	−0.002*** (0.000)
% of the population living in urban areas	−0.000 (0.004)	−0.002*** (0.000)
Participation price elasticity	−0.703**	−0.710***

Coefficients are average marginal effects. Standard errors are clustered by country and indicated in parentheses. All regressions include year-fixed effects.

*p < .1; **p < .05; ***p < .01.

More exposure to cigarette advertising is associated with higher youth smoking participation for local brands, but not foreign brands. The local rate of exposure to antismoking messages is negatively associated with smoking participation for foreign brands but has no significant effect on smoking participation for local brands. Also, youth are less likely to smoke in areas where it is harder for them to purchase cigarettes.

In terms of individual-level characteristics, we find that males have a substantially higher probability of smoking. The probability of smoking increases as age increases. The coefficient on age must be interpreted in conjunction with the coefficient on age squared. The two coefficients suggest an upward sloping parabola, which means that older ages are associated with a higher probability of smoking participation, and the probability increases at an increasing rate. Education tells a similar story, with students in grade 11 significantly more likely to participate in cigarette smoking than grade 7 students. Receiving pocket money and having at least one parent that smokes are also statistically significant predictors of smoking.

### Conditional Cigarette Demand

Results from the smoking intensity models are presented in Table 5 for local-brand prices and foreign-brand prices. Cigarette price is negatively and significantly (at the 1% level) associated with lower cigarette consumption among smokers. The price elasticity of conditional cigarette demand is −0.44 [95% CI: −0.76 to −0.12] for local brands and −0.75 [95% CI: −0.96 to −0.53] for foreign brands.

**Table 5.** Generalized Linear Model of Conditional Demand (Local- and Foreign-Brand Prices)

	Conditional demand	
	Local-brand	Foreign-brand
	(*N* = 4002)	(*N* = 3962)
Log of cigarette price	−0.444*** (0.163)	−0.746*** (0.110)
Local rate of exposure to cigarette advertising	0.099 (0.306)	0.192 (0.275)
Local rate of exposure to antismoking messages	−1.159*** (0.352)	−1.758*** (0.397)
Antismoking sentiment	−0.109 (0.179)	−0.257 (0.204)
Youth access	−0.446 (0.281)	−0.307 (0.221)
Age	−0.826** (0.388)	−0.961*** (0.323)
Age2	0.031** (0.013)	0.034*** (0.011)
Male	0.186 (0.143)	0.166 (0.160)
Pocket money	0.107 (0.071)	0.089 (0.077)
Parental smoking	0.264*** (0.104)	0.267** (0.107)
Education (relative to grade 7)		
Grade 8	0.052 (0.060)	0.068 (0.056)
Grade 9	0.007 (0.061)	0.030 (0.78)
Grade 10	0.038 (0.112)	0.058 (0.104)
Grade 11	0.287** (0.136)	0.350*** (0.127)
Poverty head count ratio	−0.009*** (0.003)	−0.080*** (0.005)
% of the population living in urban areas	−0.003 (0.003)	−0.028*** (0.007)
Conditional demand price elasticity	−0.444***	−0.746***

Standard errors are clustered by country and indicated in parentheses. All regressions include year-fixed effects.

**p < .05; ***p < .01.

We find no evidence that antismoking sentiment, cigarette advertising, or youth access influence the number of cigarettes smoked by current smokers. This suggests that once the decision to smoke is made, environmental factors other than cigarette prices are less powerful in determining how many cigarettes are smoked. One exception is antitobacco media exposure, which is shown to reduce the number of cigarettes smoked significantly. At the individual level, only parental smoking and age affect the intensity of cigarette consumption. The age effect is quite strong (and parabolic, ie, increasing at an increasing rate over the course of the age groups).

## Discussion

Our results show that higher cigarette prices are associated with reductions in smoking prevalence and cigarette consumption among youths in a sample of 16 African countries. Our results are robust to different sets of prices.

Our estimate of the total price elasticity of demand for cigarette consumption by youths is −1.147 for the local-brand specification and −1.456 for the foreign-brand specification. Since foreign brands are generally more expensive than local brands (Table 3), our finding of a higher price elasticity in the foreign-brand specification suggests that African youths may be more responsive to price increases when prices are already high to begin with.

While no comparable study using survey data has been done for adults in this group of countries, when we compare our elasticity estimates with those found for African adults in single-country studies, it is clear that youths are significantly more price responsive than their adult counterparts. In the peer-reviewed literature on the price elasticity of adult smoking *participation*, elasticities among adults range from −0.20 in the Gambia^30^ to between −0.18 and −0.29 in South Africa.^16^*Conditional* price elasticities among adults range from between −0.26 and −0.33 in Uganda,^9^ to between −0.43 and −0.69 in South Africa,^16^ and is estimated at −0.62 in Nigeria.^7^

Our sample includes two high-income countries, Mauritius and the Seychelles. It is therefore not entirely correct to compare our elasticity estimates with those obtained for samples of LMICs. Our unconditional price elasticity estimates are lower than those found by Kostova et al.^4^ and Nikaj and Chaloupka,^5^ who estimate total price elasticities of −2.1 and −2.2, respectively, in their multi-country studies of the price elasticity of demand in LMICs.

Our results also show that parental smoking significantly increases the likelihood of smoking and increases the intensity of cigarette consumption. In the context of such an intergenerational effect on smoking outcomes, tobacco taxation can serve as a particularly effective tool for reducing youth tobacco use. This is because it can decrease teenage smoking directly through the effect of price increases on smoking behavior and indirectly through altering parents’ smoking behavior. There is a substantial literature that indicates that adult smoking prevalence and smoking intensity are negatively correlated with cigarette prices.^2^

Our study has limitations. The strength of our results is limited by the lack of survey data over time and our corresponding inability to control for country-fixed effects. They therefore point only to the direction and strength of the relationship between price and smoking outcomes for youth in Africa. We are unable to make any causal inferences. Our use of pocket money reflects “access to income,” as opposed to being a proxy for personal income. Our use of one price per country does not take into consideration the reality that there is price variation within a country. The two data sources used for our measure of price in this analysis may collect prices from different retail outlets (it is not clear which retail outlets they access), thereby reducing their direct comparability.

This paper has shown that numerous factors, both demographic and policy-related, influence youth smoking prevalence and intensity. While non-price policy-related variables (eg, tobacco advertising exposure and exposure to antismoking messaging) are not consistently significant covariates of youth smoking prevalence and intensity across various model specifications, cigarette prices are. Since governments can influence the retail price of cigarettes through increased excise taxation, our study points to the need for African governments to increase excise taxes to discourage youth smoking and, ultimately, prevent the continent from becoming the future epicenter of the tobacco epidemic.

## Supplementary Material

A Contributorship Form detailing each author’s specific involvement with this content, as well as any supplementary data, are available online at https://academic.oup.com/ntr.

ntac017_suppl_Supplementary_Data

## Data Availability

Data are publicly available at: https://www.cdc.gov/tobacco/global/gtss/index.htm.
